# Effectiveness of dietary supplements for skin photoaging in healthy adults: a systematic review and meta-analysis of randomized controlled trials

**DOI:** 10.3389/fmed.2025.1582946

**Published:** 2025-07-21

**Authors:** Qifeng Yang, Haoying Li, Hanxin Zhang, Luyao Ma, Xiaofeng Zhang, Jingping Wu

**Affiliations:** ^1^Department of Medical Cosmetology, Hospital of Chengdu University of Traditional Chinese Medicine, Chengdu, China; ^2^Baoji Traditional Chinese Medicine Hospital, Baoji, China; ^3^The Fifth Hospital of Xi’an City, Xi’an, China

**Keywords:** skin photoaging, dietary supplement, MED, R2, randomized controlled trials, metaanalysis

## Abstract

**Background:**

The dietary supplement industry offers a wide range of orally consumed products that claim to combat skin photoaging, however, there is a lack of research on the proof of safety and effectiveness of dietary supplements in anti-skin photoaging. To further confirm their safety and efficacy, this article provides a detailed review and meta-analysis.

**Methods:**

Four databases, including PubMed, Embase, Web of Science, and the Cochrane Library (Central Database), were searched for relevant literature up to October 2024. A total of forty English-language randomized controlled trials (RCTs) investigating the relationship between dietary supplements and skin photoaging were screened for an in-depth review and meta-analysis.

**Results:**

Collagen, flavanols, and other polyphenol supplements have been found to alleviate skin photoaging and increase MED or overall skin elasticity (R2) when compared to a placebo. However, hyaluronic acid, lycopene, and carotenoids did not show any significant benefits in improving skin photoaging or MED/R2. Due to inconsistent findings and limited research, the effects of dietary supplements on skin photoaging could not be determined in randomized controlled trials with two or fewer studies. It is important to note that, during the study period (typically ≤ 24 weeks), all orally administered dietary supplements were found to be safe.

**Conclusion:**

Oral dietary supplements, such as collagen, flavanols, and polyphenols, have demonstrated effectiveness in addressing skin photoaging. However, there is currently insufficient evidence to support the recommendation of other dietary supplements, such as astaxanthin, for the treatment of skin photoaging. This research serves as an initial step in exploring the benefits of dietary supplements in combating skin photoaging. It underscores the need for more focused studies to further investigate the effects of dietary supplements on skin photoaging and gather additional evidence supporting their efficacy.

**Systematic review registration:**

https://www.crd.york.ac.uk/PROSPERO/view/CRD42023472473, identifier CRD42023472473.

## 1 Introduction

Enhancing the skin’s softness and aesthetics is a widespread aspiration, particularly among women. Functioning as the human body’s largest organ, the skin serves as a protective shield, separating cells from the external surroundings, while enwrapping a significant expanse of the body (1). Skin aging is an intricate process characterized by various morphological and physiological alterations that naturally occur with the passage of time and are exacerbated by environmental factors. While the interaction between genetic and environmental factors plays a significant role, the prominent mechanism of skin aging is primarily attributed to prolonged exposure to solar ultraviolet (UV) radiation, commonly known as photoaging (2, 3). Extended exposure to solar UV radiation has been associated with a range of negative impacts on the skin, including the development of wrinkles, dryness, hyperpigmentation, thinning of the topmost layer of the skin, heightened fragility, and noticeable changes in the elastic fibers of the underlying skin layer. These alterations contribute to decreased skin suppleness, weakened strength, insufficient collagen production, and ultimately, skin looseness (2, 4). Moreover, UV radiation is extensively acknowledged as a prevalent environmental carcinogen, and excessive exposure is closely associated with the development of skin cancer. The accumulating evidence strongly supports the idea that UV levels are rising due to the depletion of stratospheric ozone and the influence of climate change. This upward trend in UV radiation levels finds robust support in an expanding body of research (5–7).

A range of strategies can be implemented to enhance the skin’s resilience against solar radiation. The World Health Organization strongly advocates for the adoption of preventive measures, including wearing protective clothing, avoiding exposure during peak hours, and regularly applying sunscreen. By incorporating these proactive measures into daily routine, individuals can significantly reduce the risk of skin damage caused by excessive UV radiation. Despite these recommendations, recent research examining sunburn prevalence in the United States reveals that 34.4% of adult Americans experienced sunburn within the past year. Incidence of sunburn was found to be highest among males, non-Hispanic white individuals, younger age groups, and those from higher income brackets. Notably, only a meager one-third of adults consistently implemented sun protection practices, with females and older individuals exhibiting a higher level of compliance. Adolescents demonstrated even lower adherence rates, with 3% reporting sunburn from the previous summer, and less than 10% engaging in proactive sun protection measures. On a more positive note, approximately 69% of parents employed sunscreen for their children’s protection, while 25% resorted to umbrellas as an additional safeguard (8). In a study conducted in Canada, a considerable percentage of participants (between 40 and 48%) reported adopting diverse protective measures to reduce their exposure to solar radiation. These measures encompassed the application of sunscreen, the utilization of protective clothing, seeking out shaded areas, and refraining from direct sunlight between the hours of 11 a.m. and 4 p.m. (9). Emerging studies have demonstrated that microneedle-assisted intradermal delivery of therapeutic supplements through skin boosters can effectively ameliorate cutaneous photoaging (10).

Based on this, an increasing number of people are starting to pay attention to photoprotection through dietary means, and there is a growing interest in natural dietary supplements for preventing UV-induced damage (2, 11, 12). In such studies, these dietary supplements are believed to possess effective antioxidant (13–15) and anti-inflammatory (14, 16, 17) properties. Recently, there is emerging data suggesting that dietary supplements may be a promising dietary photoprotection approach (18–22). Furthermore, research has demonstrated that specific natural dietary supplements possess the potential to enhance the microcirculation of human skin (23, 24).

However, RCTs examining the efficacy of dietary supplements in mitigating skin photoaging have consistently returned results that are characterized by confusion, inconsistency, and potential hardships for consumers. The scientific substantiation for their purported anti-photoaging effects remains inadequate, primarily stemming from *in vitro* experiments or uncontrolled *in vivo* studies. To date, the scientific substantiation regarding the effectiveness and safety of dietary supplements in countering skin photoaging has been insufficient and unconvincing. Hence, the objective of this study was to comprehensively review existing literature on dietary supplements and their potential anti-skin photoaging benefits. Through an extensive literature search and meta-analysis of randomized controlled trials, we aimed to determine the efficacy and safety of these supplements in mitigating skin photoaging. Thus, the relationship between dietary supplements and skin photoaging can be scientifically understood and applied in daily skin care.

## 2 Methods

This article adheres to the PRISMA (Reporting Guidelines for Systematic Evaluation and Meta-analysis). The guidelines were followed in terms of accreditation, study selection, eligibility criteria, and inclusion criteria (25). The study protocol was pre-registered in the PROSPERO database, in accordance with best practices (No. CRD42023472473).

### 2.1 Search strategy

We conducted a comprehensive search for relevant literature published between January 2000 and October 2024, using four databases: Pub-Med, Embase, Web of Science, and Cochrane Library (Central Database). The search terms were 1) skin aging or skin conditions or skin photoaging or UVB and 2) dietary supplements or oral or ingestion. The search terms were tailored for each specific database, and the complete search strategy can be found in Supplementary Table S1. For this article, only RCTs conducted in English were included.

### 2.2 Eligibility criteria

A study was included in our analysis if it satisfied all the following criteria for inclusion:

(1) Study category. We only included RCTs utilizing a parallel-group design to evaluate skin conditions such as MED or Overall Skin Elasticity in human subjects. This study design allows for the control of participant variations by randomizing them into parallel groups. Furthermore, the eligible studies included a control group that received either a placebo or a low-dose supplement to account for any potential placebo effect. We limited our inclusion criteria to peer-reviewed full-text articles, and only English-language RCTs were considered for this publication.

(2) Participant characteristics. We enrolled a diverse cohort of healthy adults, spanning a wide range of ages from 18 to 75 years, and including individuals of various genders and races. Exclusion criteria were applied to ensure the absence of significant underlying health conditions among the participants: (A) We excluded individuals who were pregnant or breastfeeding, and those diagnosed with metabolic, cardiovascular, or hepatic disorders from participating in the study; (B) Subjects with significant skin-related conditions, including atopic dermatitis, psoriasis, and vitiligo, were excluded from the study; (C) Participants were asked not to have recently consumed any foods, medications, or supplements that could affect the condition of their skin. This includes items such as health foods, antioxidant supplements, retinoids, steroids, and hormonal products.

(3) Intervention strategy. Included were randomized controlled trials that compared the efficacy of dietary supplements to either placebo or lower dosages of the same supplement. To be eligible, the test and control groups were required to adhere to identical protocols, regardless of the implementation of any additional lifestyle interventions. Studies that employed divergent regimens between the intervention and comparison groups were not considered for inclusion.

### 2.3 Study selection

Three reviewers (Q.Y., J.W., or H.L.) autonomously analyzed titles and abstracts to identify relevant studies. Full-text articles were imported into Endnote software (version X9, Clarivate Analytics) for further evaluation only if they met the criteria for potential eligibility during the initial screening. The reviewers (H.L., H.Z., and X.Z.) separately scrutinized the full texts using the pre-established inclusion criteria to determine their suitability for the study. In cases of uncertainty, the reviewers consulted relevant literature or experts (Q.Y.,H.Z. and L.M.) for clarification.

### 2.4 Data extraction

Supplementary Table S2 documented the specific characteristics of each study that was included in the analysis. This table offered in-depth information regarding various aspects of the studies, encompassing details such as the author(s), publication year, country of origin, participant demographics (including age, gender, and health status), study duration, intervention specifics for each group, daily dosage, application method, environmental parameters (room temperature in degrees Celsius and relative humidity percentage), as well as the specific area of the skin that was evaluated. To determine the safety of the interventions, a comparison was made between the intervention and placebo groups’ adverse events (AEs), treatment-emergent adverse events (TEA), and treatment-associated withdrawals (TAW).

All other data collected, including baseline and endpoint measurements (mean) and their corresponding measures of variability (standard deviation (SD) or standard error (SE)), were meticulously recorded in an Excel spreadsheet. The study focused on analyzing two specific outcomes: MED and overall skin elasticity. When the study provided data from multiple measurement sites, the face and arm test areas, which are of high concern to women, were preferred as measurement sites. For some studies with outcome measures at multiple time points, data were extracted for the longest observation duration.

Whenever there were any problems during the data collection process, another researcher was consulted to negotiate a solution.

### 2.5 Quality assessment

It is necessary to assess the following potential biases in the study, such as selection bias (SB), implementation bias (PB), detection bias (DB), attrition bias (AB), reporting bias (RB), and other biases related to the experimental structure or approach factors (26). Possible sources of other biases - including changes in participants’ lifestyle habits, unstable testing conditions that may affect measurement results, or conflicts of interest - were also evaluated. Each study was assessed for each criterion and labeled as having “low,” “high,” or “unclear” risk of bias. A “low risk of bias” was considered when the study reported methods that were in line with the Cochrane Collaboration’s guidelines and could reduce potential biases. Conversely, a “high risk of bias” was assigned when the study employed methods that the potential biases could not be eradicated. If there was insufficient information or inadequate reporting, the trial was labeled as having “unclear risk of bias” (26).

### 2.6 Statistical analysis

#### 2.6.1 Evaluation of overall effect size

A total of 22 RCTs were included in the analysis and subjected to meta-analysis using Review Manager 5.4. The random effects model was applied in the statistical analysis to calculate the standardized mean differences (SMDs) for continuous variables. The mean difference was derived by subtracting the baseline value from the endpoint value. In cases where changes in standard deviations (SDs) from baseline were not reported, the SDs for the treatment and placebo groups were estimated using the following formula based on the SDs at baseline and final time points:


$$S\,D=\sqrt{\left(S\,D\,\_\,b\,a\,s\,e\,l\,i\,n\,e^{\wedge }\,2+S\,D\,\_\,f\,i\,n\,a\,l^{\wedge }\,2\right)-2\,x\,C\,o\,r\,r\,x\,S\,D\,\_\,b\,a\,s\,e\,l\,i\,n\,e\,x\,S\,D\,\_\,f\,i\,n\,a\,l}$$

Furthermore, if the RCT lacked a placebo group for comparison and only assessed the effects of different dosage levels of the intervention, the high-dose group was treated as the experimental group, while the low-dose group served as the control group. Due to the absence of reported baseline-endpoint correlation coefficients (Corr), a standard Corr value of 0.5 was employed. This value is commonly utilized in studies with sufficient data and typically falls within the range of 0.4–0.6.

The minimum erythema dose (MED) and overall skin elasticity were quantified using the standard mean difference (SMD) with a 95% confidence interval (CI). This was necessary because the included studies did not consistently report these six outcomes using the same measurement device and unit. A *p*-value of less than 0.05 was considered statistically significant (27).

#### 2.6.2 Evaluation of heterogeneity

The forest plots in this study include notes indicating the measure of heterogeneity statistics, estimated using Higgins I (I^2^). This index denotes the proportion of differences in effect sizes that can be attributed to variability across studies, rather than sampling error (28). A value of 0% for I^2^ suggests that the variance in effect size is solely due to sampling error. Conversely, a value of 100% suggests that variability across studies explains the entire variance in effect size. Typically, I^2^ values within the ranges of 0–25, 25–50, 50–75, and 75–100% correspond to low, moderate, high, and very high levels of heterogeneity, respectively (29).

## 3 Results

### 3.1 Identification of researches

**FIGURE 1:**
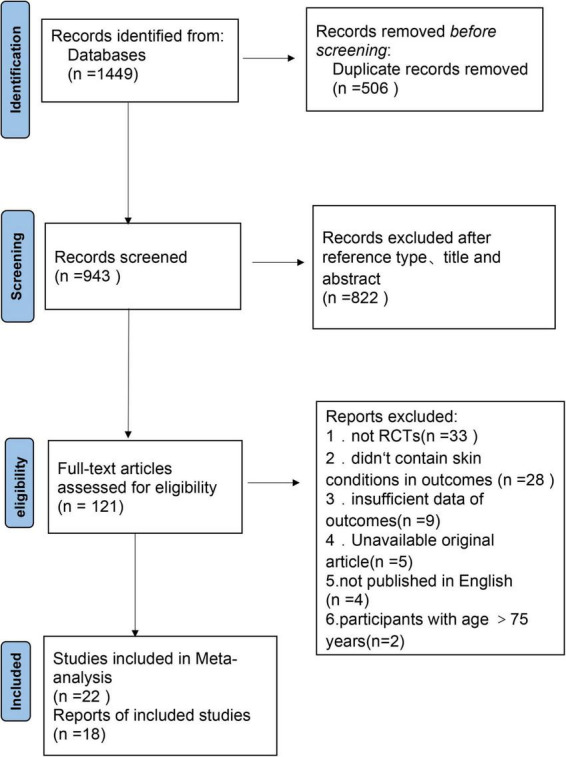
PRISMA diagram of included studies.

A comprehensive search yielded a total of 1,449 articles (Figure 1). These comprised 344 articles from PubMed, 515 articles from Embase, 314 articles from Web of Science, and 276 articles from the Cochrane Library’s Central Database. After a thorough screening process, 40 randomized controlled trials were ultimately selected for inclusion in the systematic review and meta-analysis.

### 3.2 Risk of bias within research

**FIGURE 2:**
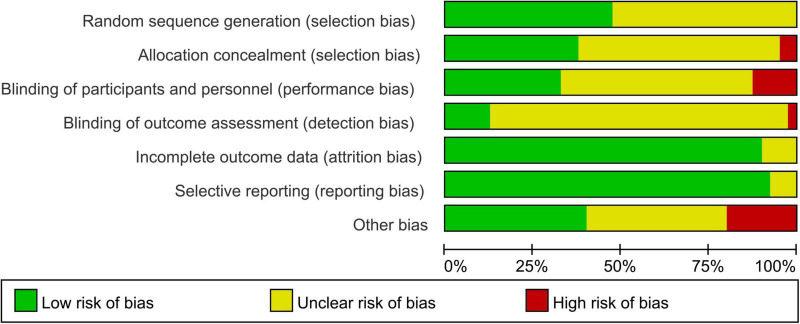
Summary of review authors’ judgements for each risk of bias domain.

Figure 2 presented the distribution of each ROB item as percentages across all the selected studies, based on the researcher’s judgment. The specific judgments of bias for each individual study are appear in Supplementary Figure S1.

Most of the trials included in the review provided limited details about their research designs and methods. As a result, the risk of Selection Bias (SB) was predominantly unknown, with only 19 out of 40 RCTs (47%) describing their randomization process in sufficient detail, and 21 studies were considered to have unclear risk. Adequate allocation concealment was reported in only 15 RCTs (38%), and 2 were identified as high-risk. Additionally, Performance Bias (PB) was largely unclear (55%), with only 22 studies acknowledging whether subjects/researchers were blinded, and 5 studies were identified as high-risk. Detection Bias (DB) was also largely unclear (83%), with only 5 studies identified as low-risk and 1 as high-risk. However, Attrition Bias (AB) was generally low, with only 3 studies regarded as having unclear risk out of 36 studies (90%). Reporting bias was unclear in only 3 studies (8%), while 37 studies (92%) provided clear data. Eight studies (21%) were rated as having a high risk of “other biases,” while 16 studies were rated as having unclear risk. Common causes for these biases included potential conflicts of interest, control of participants’ living habits, and insufficient information on physical environmental conditions during hydration measurement. The observed selection bias (SB) and performance bias (PB) were primarily attributed to insufficient methodological documentation in included studies, particularly regarding inadequate description of randomization procedures and implementation of blinding protocols for both investigators and participants. This methodological ambiguity may consequently lead to compromised overall study homogeneity and potential susceptibility to performance bias effects.

### 3.3 Research features

In Supplementary Table S2, an overview of the main characteristics of each study is provided. The most commonly utilized dietary supplements comprised collagen (*n* = 14, 35%), flavanols (*n* = 5, 12.5%), polyphenols (*n* = 5, 12.5%), carotenoids (*n* = 5, 12.5%), hyaluronic acid (*n* = 4, 10%), lycopene (*n* = 4, 10%), and astaxanthin (*n* = 3, 7.5%). Furthermore, a comprehensive meta-analysis was performed, specifically analyzing six groups consisting of 22 out of the total 40 studies. The objective was to assess the impact of oral dietary supplements on skin photoaging. The collagen group collected eight studies involving Asians (Korean, Japanese, Chinese) and Europeans (Germans and Italians), and therefore performed an ethnic subgroup analysis with differences between Asians and Europeans in all measured outcome indicators. The study primarily included women ranging from 20 to 75 years old who exhibited signs of skin photoaging, such as sunspots and reduced skin elasticity. The trial involved the administration of capsules, beverages, and tablets containing the test product or a placebo. The duration of the majority of trials ranged from 4 to 12 weeks. Furthermore, to ensure accurate measurements, the research efforts focused on average values of relative temperature (22°C) and relative humidity (50%) as crucial environmental conditions.

### 3.4 Collagen

A total of 14 studies (*n* = 888) were conducted to explore the effects of collagen supplementation. A meta-analysis was performed on 8 of these studies (*n* = 306), including 5 studies (30–34) with an Asian population (*n* = 291) and 3 studies (35–37) with a European population (*n* = 312). The research encompassed a range of formulations including capsules, tablets, beverages, and powders. These formulations contained collagen peptides or hydrolysates with molecular weights varying from 10 to 5,000 mg. Each of the 14 studies (30, 32–44) included a placebo group with the same composition as the intervention group, excluding the active ingredients. Study periods varied from 4 to 12 weeks in length.

**FIGURE 3:**
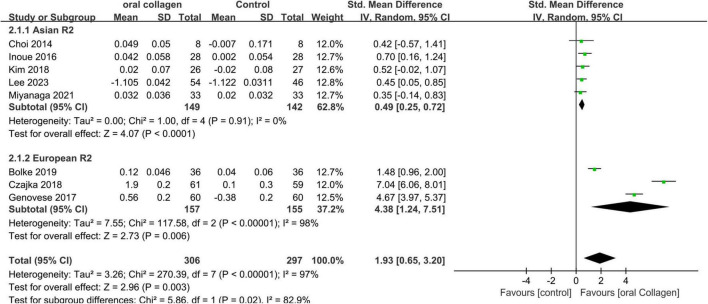
Forest plot of comparison: Collagen vs. placebo on Overall skin elasticity(R2) (SMD). The details of each study are reported in Supplementary Table S2. CI, confidence interval.

The key data extracted primarily focuses on the R2. Figure 3 presents a forest plot depicting a meta-analysis conducted on eight studies, aiming to estimate the disparity in R2 values between the experimental and control groups.

A meta-analysis of 8 studies involving 306 participants showed significant differences in the consumption of collagen-based dietary supplements compared to placebo groups in both Asian (SMD = 0.49; 95% CI: 0.25–0.72; *p* < 0.0001) and European cohorts (SMD = 4.38; 95% CI: 1.24–7.51; *p* = 0.006). Furthermore, an overall effect size pooled analysis (SMD = 1.93; 95% CI: 0.65–3.20; *p* = 0.003) provided statistical evidence supporting that collagen intake increases R2, indicating the effectiveness of collagen dietary supplementation against dermal photodamage as a form of skin aging. In the Asian cohort, no heterogeneity was observed (I^2^ = 0%). However, high heterogeneity was noted in the analysis of the European cohort (I^2^ = 98%). Sensitivity analysis through one-by-one exclusion indicated that the source of heterogeneity could not be identified, possibly due to variations in dietary supplement formulations or uncontrolled measurement environments among the included populations.

Based on the 14 studies included in the review (30, 32–43), safety was addressed in 12 of them (30–37, 39–41, 43). Among these, 11 studies (30–37, 40, 41, 43) reported no adverse events (AEs), while one study (39) reported an AE without any associated serious or treatment-related adverse events (TAEs). However, the original manuscript lacks comprehensive documentation regarding adverse events, including critical details such as incidence rates, severity grading.

### 3.5 Flavanol

Flavanols were tested in five studies (18–22) (*n* = 191) over a period of 1–24 weeks in dosage forms ranging from capsules, beverages, and chocolates, and the molecular weight of the active ingredients involved were mostly flavanols and related preparations with molecular weights ranging from 55 to 600 mg. Four of the five studies (18–20, 22) had a low dose group as a control, and one (21) had a placebo group with a composition identical to the experimental composition, except for the active ingredient. placebo group consistent with the composition of the group.

The data related to MED and R2 were extracted from the studies, enabling a meta-analysis of the five studies to be conducted in two groups.

#### 3.5.1 Minimum erythema dose

In four studies (18, 20–22) involving a total of 167 participants, the MED was measured. One study focused on a combination formulation (18), which primarily included active components such as polyphenols, total flavanols, epicatechin, and catechin, administered at a dosage of 4–6 g. The remaining three studies investigated individual flavanol components (20–22) at doses ranging from 200 to 600 mg flavanols. In three of the four studies, a low flavanol group was used as the control group (18, 20, 22), while one study employed a placebo group with an equivalent dosage and other nutritional substances (21). The changes in MED were reported after the consumption of flavanols for 1–24 weeks.

**FIGURE 4:**
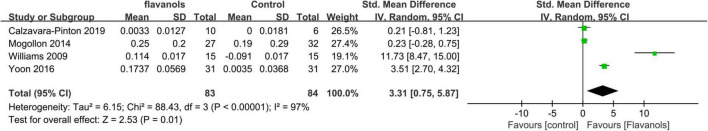
Forest plot of comparison: Flavanols vs. placebo on MED(SMD). The details of each study are reported in Supplementary Table S2. CI, confidence interval.

Figure 4 presents the forest plot of a meta-analysis comprising four studies, which assesses the disparity in MED between experimental and control groups.

Statistical analysis demonstrated a significant difference in MED when comparing the effects of high-flavanol ingestion to both low-flavanol and placebo (SMD = 3.31;95% CI:0.75–5.87; *p* = 0.01) (Figure 4), indicating that flavanol consumption effectively prolongs MED. However, meta-analysis revealed high heterogeneity (I^2^ = 97%), Sensitivity analysis through one-by-one exclusion indicated that the source of heterogeneity could not be identified, potentially due to differences in administered flavanol formulations and variations in measurement sites across the studies.

#### 3.5.2 Overall skin elasticity(R2)

Overall skin elasticity was evaluated in two studies (21, 22) consisting of 121 participants for 12–24 weeks. Both studies focused on a single flavanol component with intake ranging from 320 to 600 mg. One study used low-flavanol as the control group (22) while the other employed a placebo group (21).

**FIGURE 5:**
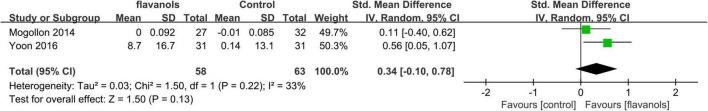
Forest plot of comparison: Flavanols vs. placebo on Overall skin elasticity(R2) (SMD). The details of each study are reported in Supplementary Table S2. CI, confidence interval.

Figure 5 displays the forest plot illustrating a meta-analysis of two studies that evaluate the difference in overall skin elasticity between the experimental and control groups.

In the meta-analysis of two studies involving a total of 121 participants, it was found that the intake of high-flavanol or flavanol did not significantly improve skin elasticity when compared to low-flavanol or placebo groups (SMD = 0.34; 95% CI: –0.10–0.78; *p* = 0.13) as demonstrated in Figure 5. The moderate heterogeneity (I^2^ = 33%) observed in the meta-analysis could be attributed to differences in the form of flavanol intake (beverage or chocolate) and variations in the body sites examined (facial, arm, and buttock).

Out of the five studies that investigated the effects of flavanols, three studies made reference to safety. In all three studies, no adverse events were reported.

### 3.6 Other polyphenols

Other polyphenols were tested in 5 studies (44–47) (*n* = 258), with one study considered as two independent experiments due to the inclusion of two ethnic groups. Among these studies, meta-analysis was conducted in 3 of them (45, 46) (*n* = 160), focusing on the efficacy of green tea polyphenols and other plant polyphenols as active compounds. The dosage of polyphenols was specified in all three studies, ranging from 250 to 1,400 mg per day over a 12-week period. Placebo groups were included in all three studies.

**FIGURE 6:**
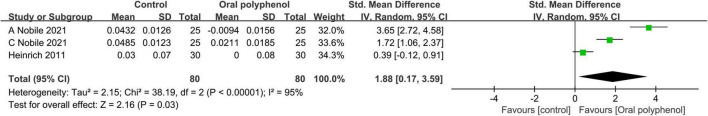
Forest plot of comparison: Polyphenols vs. placebo on Overall skin elasticity(R2) (SMD). The details of each study are reported in Supplementary Table S2. CI, confidence interval.

The primary data extracted from three studies primarily centered on R2. Figure 6 presents a forest plot representing a meta-analysis of these three studies, demonstrating the estimated difference in R2 between the experimental and control groups.

The meta-analysis of three studies revealed a positive impact of polyphenol dietary supplements on improving R2 (SMD = 1.88; 95% CI: 0.17–3.59; *p* = 0.03) as shown in Figure 6. These supplements showed effective improvement in photoaging. However, it is important to note that a high level of heterogeneity was observed (I^2^ = 95%). Sensitivity analysis through one-by-one exclusion indicated that the source of heterogeneity could not be identified, This phenomenon may be attributed to variations in ethnicity, ambient temperature, or the Measuring Sites.

Safety was mentioned in four of the five studies (44, 45, 47), all of which reported no AE.

### 3.7 Lycopene

A total of 4 studies (48–51) (*n* = 306) investigated the efficacy of lycopene, with meta-analyses conducted on 2 studies (48, 49). The studies included various formulations such as capsules and pastes, with lycopene doses ranging from 10 to 16 mg. In all 4 studies (48–51), a placebo group was included, which had similar composition to the experimental group except for the active ingredient. Study durations varied from 10 to 12 weeks.

**FIGURE 7:**
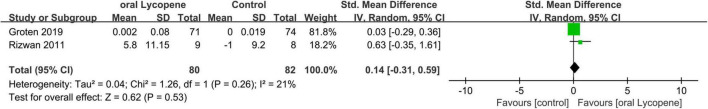
Forest plot of comparison: lycopene vs. placebo on Overall skin elasticity(R2) (SMD). The details of each study are reported in Supplementary Table S2. CI, confidence interval.

Data on R2 primarily originated from two studies. Figure 7 displays a forest plot visualizing the meta-analysis of these two studies. The forest plot provides an estimation of the disparity in R2 between the experimental and control groups.

The meta-analysis results indicated that the experimental group did not achieve a significant improvement in R2 compared to the placebo group (SMD = 0.14; 95% CI: –0.31–0.59, *p* = 0.53) (I^2^ = 21%). (Figure 7). That is, intake of lycopene dietary supplements had no beneficial effect on improving skin photoaging in this study.

Three out of four studies (48, 49, 51) provided safety reports, and none of them detected any adverse events during the treatment period.

### 3.8 Carotenoids

Over a period of 6–12 weeks, five studies (52–56) with 196 participants investigated the use of carotenoids as dietary supplements. A meta-analysis of two of these studies (53, 54), which involved 71 participants taking capsules or tablets, was con-ducted. One study (54) used a low-dose group as the control, while the other (53) used a placebo group for comparison.

**FIGURE 8:**
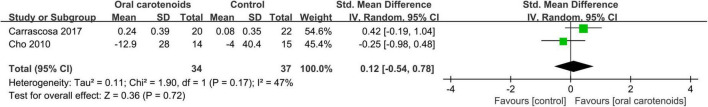
Forest plot of comparison: Carotenoids vs. placebo on Overall skin elasticity(R2) (SMD). The details of each study are reported in Supplementary Table S2. CI, confidence interval.

The forest plot in Figure 8 presents a meta-analysis of two studies, providing an estimation of the discrepancy in R2 between the experimental and control groups.

The meta-analysis findings revealed no statistically significant distinction between the intervention and placebo groups in terms of Overall skin elasticity (SMD = 0.12; 95% CI: –0.54–0.78, *p* = 0.72) (I^2^ = 47%) (Figure 8). In the paper, it indicates that the ingestion of carotenoids does not show any improvement in skin photoaging.

Safety was mentioned in five studies, none of which reported the occurrence of adverse events.

### 3.9 Hyaluronan

Four studies (57–60) with a total of 178 participants explored the use of hyaluronic acid. A meta-analysis was conducted on two of these studies (57, 58), involving 100 participants. The interventions in these studies included hyaluronic acid formulations in the form of capsules or beverages, with the effective ingredient doses ranging from 120 to 200 mg. All four studies included a placebo group that had the same composition as the experimental group, except for the active ingredient. The studies exhibited a varying duration, with a range of 4–12 weeks.

**FIGURE 9:**
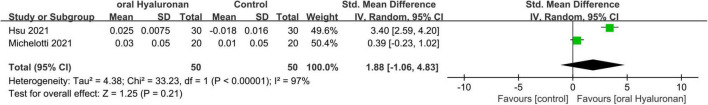
Forest plot of comparison: Hyaluronan vs. placebo on Overall skin elasticity(R2) (SMD). The details of each study are reported in Supplementary Table S2. CI, confidence interval.

As extracted from two studies, relevant data on R2 were mainly obtained. Figure 9 presents a forest plot displaying the outcome of a me-ta-analysis on the difference of R2 observed between the intervention and placebo groups in these studies.

The Meta-analysis revealed no significant statistically discernable distinction between the group that received hyaluronic acid and the group that received a placebo. (SMD = 1.88;95% CI: –1.06–4.83, *p* = 0.21) ((I^2^ = 97%).)(Figure 9). The two studies exhibited substantial heterogeneity, potentially attributable to differences in anatomical measurement sites and variations in ethnic or gender composition among study populations. This indicates that the intake of hyaluronic acid does not improve photoaging.

Three of the four studies (57–59) explicitly did not have adverse events, and one study (60) did not mention safety.

### 3.10 Astaxanthin

Three studies (61–63) (*n* = 102) investigated the effects of astaxanthin with dosages ranging from 2 to 4 mg, all administered in capsule form. Each study included a placebo group with the same composition as the experimental group, except for the active ingredient. The time span of the study was between 6 and 12 weeks. Two studies (61, 62) mentioned the safety of astaxanthin and reported no adverse events.

## 4 Discussion

Using a systematic review and meta-analysis approach, this study investigated the effects of dietary supplements on photoaging of the skin. A total of 40 randomized controlled trials with 2,119 healthy participants were included in the analysis. To evaluate the impact of dietary supplements on skin photoaging, this study measured the MED and R2 as indicators. It is worth mentioning that no previous systematic review or meta-analysis has been published on the subject of using dietary supplements to combat skin photoaging. This study addresses the existing lacuna in research by conducting an in-depth analysis and meta-analysis, which presents updated supporting material regarding the safety and effectiveness of dietary supplements in the treatment of skin photoaging.

### 4.1 Oral dietary supplement effects on skin photoaging

The studies analyzed in this research focused on two main outcome measures: MED and R2. MED is a measure of the minimum amount of ultraviolet radiation (expressed in j/cm2), or the shortest exposure time needed to induce the least visible redness on the skin, known as erythema. The MED value varies depending on each individual’s skin photosensitivity, also known as phototype. MED is a key measure utilized to evaluate the impact of skin photoaging. A higher MED value indicates a greater ability to combat photoaging of the skin (64). In addition, R2 is an essential indicator of skin aging, providing a comprehensive assessment of skin elasticity status.

**FIGURE 10:**
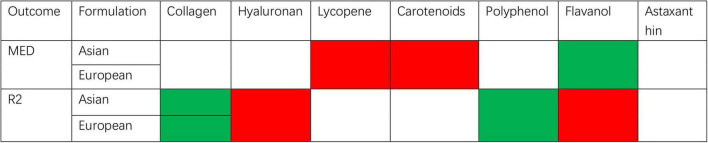
Summary of meta-analysis of studies reporting the effect of dietary supplements on skin moisturizing.

This meta-analysis is represented in Figure 10 and illustrates the effects of dietary supplements on photoaging. Flavanols, all derived from cocoa, were independently analyzed and reported due to their distinct characteristics. The inclusion of polyphenols and collagen-based dietary supplements demonstrated an improvement in R2, while only flavanols significantly enhanced MED. No significant effects on any of the outcome measures of photoaging were observed for lycopene, carotenoids, and hyaluronic ac-id-based dietary supplements. In the collagen group, eight studies were included with low heterogeneity and low risk for quality assessment of the literature, making the analysis more reliable. The five studies included in the cocoa flavanols group had high heterogeneity and the source of heterogeneity was unclear, so more cutting-edge, high-quality literature would need to be included in the analysis if further reliable evidence is to be obtained. The three studies included in the other polyphenol group also had high heterogeneity and obtained less reliable results. For the data analysis in the literature, only two studies were included in the hyaluronic acid group, lycopene group, and carotenoid group. In order to obtain more reliable results, further attention should be paid to relevant cutting-edge disciplines and more research data should be included for analysis. Therefore, based on the analysis of this study, collagen dietary supplements have been found to be effective against photoaging. Cocoa flavanols and other poly-phenol dietary supplements also show potential effectiveness against photoaging, but the evidence is not yet reliable. The findings of such trials could have significant clinical implications for the management and prevention of skin aging, as well as for the development of new supplement therapeutics. Oral nutraceutical supplementation represents a clinically relevant intervention for ameliorating the adverse effects of cutaneous photoaging, particularly in individuals with contraindications to topical sunscreens or physical photoprotection measures. This approach also demonstrates therapeutic value in post-sunburn recovery, where adjunctive topical treatments can synergistically enhance tissue repair and accelerate the reversal of photoaging damage. This highlights the importance of continued research in this field to further advance our understanding of the potential benefits of dietary supplements for skin health.

However, it is important to note that this conclusion is based on a limited amount of data and that more studies are necessary to confirm the safety of these supplements. It is also crucial to carefully monitor potential adverse reactions and ensure that the benefits of these supplements outweigh any potential risks. In summary, while the existing data suggests safety, further research is needed to solidify these findings and establish guidelines for the safe use of dietary supplements in the context of skin photoaging.

### 4.2 Mechanism

While some dietary supplements have been used in related skin care, the exact underlying causal relationship and specific mechanisms of dietary supplements against skin photoaging remain elusive.

Collagen, an essential biomolecule, plays a critical role in animal connective tissues as the primary building block. It is the most abundant and widely distributed functional protein found throughout the bodies of mammals. Constituting approximately 25–30% of the total protein content, collagen can even reach extraordinarily high levels, exceeding 80%, in select organisms (65). One potential explanation for the positive impact of orally ingested collagen on skin photoaging is that collagen constitutes a crucial structural protein within the skin. This protein offers support to retain skin elasticity and moisture content, thereby contributing to healthier skin function (66). By supplementing the required collagen in the skin, oral intake of collagen can enhance skin moisturizing ability, promote the regeneration of skin cells, accelerate skin metabolism, and make the skin more elastic and glossy (67). In addition, collagen is a rich source of amino acids, including hydroxyproline which is a major component of collagen. Hydroxyproline possesses potent antioxidant properties that can effectively counteract free radical damage and prevent the adverse effects of skin photoaging (68). As collagen in the skin is continuously degraded with age, leading to skin relaxation and wrinkles, oral intake of collagen can slow down this degradation process, improve skin elasticity and firmness (33).

Polyphenols are secondary metabolites widely present in plants and are potent antioxidants (69, 70). They can neutralize free radicals and reduce oxidative damage to the skin. Excessive production of free radicals can lead to oxidative damage and aging of skin cells, while the antioxidant properties of polyphenols can protect the skin from oxidative stress (71–74). Furthermore, polyphenols have demonstrated anti-inflammatory properties that can help to mitigate skin inflammation and enhance the synthesis and manufacture of collagen within the skin (75–78). As a key structural protein, collagen plays a pivotal role in maintaining optimal skin elasticity and promoting its natural radiance. Some polyphenols also exhibit inhibitory effects on melanin production, reducing issues such as hyperpigmentation, freckles, and pigmentation, resulting in a more even and brighter complexion (79, 80). Additionally, polyphenols enhance the functionality of the skin barrier, reducing moisture loss and improving skin hydration. They help moisturize and nourish the skin, addressing problems such as dryness, roughness, and loss of elasticity.

Flavonoids are a significant type of polyphenol, which can be further classified into subcategories based on their heterocyclic structure and substituents. These subcategories comprise flavanones, flavanols, flavones, isoflavones, and anthocyanins (81). Therefore, cocoa flavanols share similarities with polyphenols in their ability to counteract photoaging. They also exhibit more pronounced effects in terms of antioxidant properties (82–84), improvement of blood circulation (24), protection of skin barrier, and anti-inflammatory activities.

A possible mechanism for the effect of other dietary supplements in this study, such as carotenoids, on photoaging is that carotenoids absorb UV rays (85) and can provide a degree of photoprotection. UV rays are widely acknowledged as a key catalyst in the process of skin photoaging, and carotenoids are able to absorb UV rays, reduce damage to the skin, and decrease the degree of blotchiness, wrinkles, and dryness of the skin. Lycopene inhibits the activity of some enzymes associated with photoaging (86), for example, tyrosinase activity. The activity of these enzymes leads to problems such as hyperpigmentation and oxidative damage to skin cells (87), and the inhibitory effect of lycopene reduces these adverse effects and improves photoaging of the skin (86). Hyaluronic acid possesses the remarkable ability to restore the impaired function of the skin barrier (88). The skin barrier, a crucial defense layer of the skin, maintains a balance of moisture and nutrients and prevents the invasion of harmful substances from the external environment (89). By enhancing the skin barrier function, oral intake of hyaluronic acid can improve the skin’s protective ability (88), reduce sensitivity to environmental stimuli, and lower the risk of photoaging. Astaxanthin disrupts the activity of enzymes such as peroxidase, flavin light synthase, and tyrosinase (90), thereby reducing melanin deposition and the formation of pigmented spots. Melanin deposition is another contributing factor to skin photoaging (91), and the inhibitory effect of astaxanthin can alleviate these adverse re-actions and improve the photoaging condition of the skin.

In summary, the specific mechanisms by which dietary supplements combat photoaging of the skin are complex, but the supplements mentioned above share a common feature of directly or indirectly replenishing nutrients to healthy skin, thereby repairing the skin’s barriers and functions, and achieving improvements in skin MED and skin elasticity.

### 4.3 Limitations

It must be acknowledged that, despite our comprehensive search approach, it is still possible that some clinical trials may have been missed. However, our ordered and comprehensive methodology should discern the majority of relevant experiments and minimize bias. Additionally, our quality examination and statistical analysis were based on the reported information and data from the included studies, rather than direct analysis of the original research. It is undeniable that our study confirms the efficacy of certain dietary supplements in improving skin photoaging. However, it is essential to acknowledge the limitation of a restricted number of included trials in our analysis. Moreover, factors such as regional climate variations may exert an influence on the observed outcomes. Therefore, to enhance the credibility and robustness of the results, it is essential to conduct further research validation with a larger and more diverse sample size. Additionally, employing a rigorous and objective methodology will be crucial in ensuring the reliability of the findings. By doing so, we can obtain more trustworthy and conclusive results in the field.

Among the 40 RCTs investigated, 28 studies reported a zero incidence of adverse events (AEs). Only one study noted the occurrence of an adverse reaction unrelated to the research, while the remaining trials did not provide any information regarding AEs, thus mitigating potential safety concerns. These findings highlight the insufficiency of important information on dietary supplements and sources in some trials, impeding a comprehensive comparison of trial effectiveness. Experts studying such products should give more importance to emphasis on reporting and addressing AEs to ensure the integrity and accuracy of research outcomes.

## 5 Conclusion

Certain dietary supplements have shown significant effects in combating skin photoaging in some aspects compared to placebos. However, their beneficial effects are still limited in other areas. More extensive and stringent studies are needed to investigate the impact of dietary supplements, especially those containing hyaluronic acid, carotenoids, lycopene, astaxanthin, and lesser-studied supplements that have only been reported on in one study. Larger-scale investigations are necessary to validate the effects of these supplements on skin aging. Rigorous methodology is crucial in the field of dietary supplement research, considering the reliance of many consumers on these products for potential health benefits. Researchers should strive for methodological rigor, adhere to CONSORT guidelines, and prioritize standardized reporting to enhance the credibility and generalizability of their findings. This will enable more informed advice and decision-making in the medical field, ultimately benefiting patients and society as a whole (92). We have the confidence that as the importance of skin photoaging continues to be recognized and as more trials are conducted, an increasing amount of beneficial evidence for dietary supplements in addressing skin photoaging will emerge.

Currently, certain orally administered dietary supplements, such as collagen, flavanols, and other polyphenols, have demonstrated efficacy in addressing skin photoaging. However, the existing evidence on the efficacy of dietary supplements like carotenoids, lycopene, and astaxanthin in mitigating skin photoaging is still limited. Given the limited number of studies and potential methodological limitations, the current evidence may not fully elucidate the effects of these supplements (e.g., carotenoids, lycopene, and astaxanthin) on photoaging. Thus, the findings of this study should not be interpreted as conclusive evidence against their potential benefits. Future rigorously designed, hypothesis-driven studies may provide further insights into their therapeutic efficacy.

## Data Availability

The original contributions presented in the study are included in the article/Supplementary material, further inquiries can be directed to the corresponding authors.
