# Supplementary material for: Effectiveness of dietary supplements for skin photoaging in healthy adults: a systematic review and meta-analysis of randomized controlled trials
Source: Front Med (Lausanne). 2025 Jul 21;12:1582946. doi: 10.3389/fmed.2025.1582946 (PMC12318760; doi:10.3389/fmed.2025.1582946)
Supplement: Supplementary file 1 [file Data_Sheet_1.pdf]

**Supplementary Material**

**Supplemental Table S1**. Search strategy used for this review.

| Database | Search strategy | Number of results |
|---|---|---|
| Pubmed | (((((((((((((((((((((((((((((skin aging[MeSH Terms]) OR (skin care[MeSH Terms])) OR (skin condition[Title/Abstract])) OR (skin barrier[Title/Abstract]))OR (Aging, Skin[Title/Abstract])) OR (Solar Aging of Skin[Title/Abstract])) OR (Photoaging of Skin[Title/Abstract])) OR (Skin Wrinkling[Title/Abstract])) OR (Skin Wrinklings[Title/Abstract])) OR (UVB[Title/Abstract])) OR (abnormalities, skin[MeSH Terms])) OR (anti-ageing[Title/Abstract])) OR (photoaged facial skin[Title/Abstract])) OR (skin health[Title/Abstract]))))) AND (((((((((((((((((dietary supplements[MeSH Terms]) OR (additive, food[MeSH Terms])) OR (diet[MeSH Terms])) OR (food[MeSH Terms])) OR (supplement[Title/Abstract])) OR (oral[Title/Abstract])) OR (intake[Title/Abstract])) OR (ingestion[Title/Abstract])) OR (dietary[Title/Abstract])) OR (Consumption[Title/Abstract])) OR (functional food[Title/Abstract])) OR (orally[Title/Abstract]))))))))) AND (((((((((randomized controlled trial[Publication Type]) OR (controlled clinical trial[Publication Type])) OR (randomized[Title/Abstract])) OR (randomly[Title/Abstract])) OR (clinical trials as topic[Title/Abstract])) OR (clinical study[Title/Abstract]) AND (clinicaltrial[Filter] OR randomized controlled trial[Filter]))))))) NOT (((((Meta-Analysis[Publication Type]) OR (Systematic Review[Publication Type])) OR (Review[Publication Type]))))))) NOT ((((mice[MeSH Terms]) OR (rats[MeSH Terms]))))) **Filters:** from 2000 – 2023 | 344 |

| | | |
|---|---|---|
| Embase | 'aging skin':ti,ab,kw OR 'senile skin':ti,ab,kw OR 'skin aging':ti,ab,kw OR 'solar aging of skin':ti,ab,kw OR 'ultraviolet b radiation'/exp OR uvb:ti,ab,kw AND 'dietary supplement'/exp OR 'diet supplement':ti,ab,kw OR 'dietary supplements':ti,ab,kw OR 'food supplement':ti,ab,kw OR 'food'/exp OR 'diet'/exp OR 'supplementary diet':ti,ab,kw OR 'diet additive':ti,ab,kw OR oral:ti,ab,kw OR intake:ti,ab,kw OR 'ingestion'/exp OR dietary:ti,ab,kw OR consumption:ti,ab,kw OR 'functional food'/exp OR 'functional foods':ti,ab,kw AND 'randomized controlled trial':de OR 'controlled clinical trial':de OR 'randomized':ti,ab OR 'placebo':ti,ab OR 'clinical trials' OR 'randomly':ti,ab OR 'clinical study':ti,ab OR 'volunteers':ti,ab AND 'human'/exp OR 'homo sapiens':ti,ab,kw OR 'human being':ti,ab,kw OR 'human body':ti,ab,kw OR 'human race':ti,ab,kw OR humans:ti,ab | 515 |
| Cochrane | MeSH descriptor: [Skin Aging] explode all trees OR (Solar Aging of Skin):ti,ab,kw OR (Photoaging of Skin):ti,ab,kw OR (Wrinkling):ti,ab,kw OR (Skin Wrinklings):ti,ab,kw OR (Skin Wrinkling):ti,ab,kw OR (Aging, Skin):ti,ab,kw AND MeSH descriptor: [Dietary Supplements] explode all trees OR MeSH descriptor: [Food] explode all trees OR MeSH descriptor: [Diet] explode all trees OR MeSH descriptor: [Functional Food] explode all trees OR (intake):ti,ab,kw OR (dietary):ti,ab,kw OR (diets):ti,ab,kw OR(ingestion):ti,ab,kw OR (consumption):ti,ab,kw OR (functional foods):ti,ab,kw | 276 |
| Web of science | (TS=(skin aging) OR TS=(skin care) OR TS=(skin condition*) OR TS=(skin barrier)OR TS=(Wrinkle*) OR TS=(Solar Aging of Skin) OR TS=(Photoaging of Skin) OR TS=(UVB*) OR TS=(anti-ageing) OR TS=(photoaged facial skin) OR TS=(skin photoprotection) OR TS=(Photo-Aged ) OR TS=(UV light) OR TS=(Photoprotective)) AND(TS=(dietary supplements)OR TS=(food) OR TS=(diet)OR TS=(supplement)OR TS=(diets) OR TS=(oral) OR TS=(intake) OR TS=(ingestion) OR TS=(dietary) OR TS=(Consumption) )AND ((((TS=(clinical trials)) OR TS=(clinical trials)) OR TS=(clinical study)) OR TS=(randomized controlled trial)) OR TS=(RCTS) | 314 |

**Supplement Table S2.** Full details of all studies included in systematic review.

| Study Location | Study Population | | | Intervention | Control | Formulation | Study Duration | Test Conditions (°C R.T.) (% R.H.) | Parameter (Measuring Sites) Unit | Adverse Effects |
|---|---|---|---|---|---|---|---|---|---|---|
| | **Sample size, sex** | **Age, years** | **Health Condition** | **Contents, daily dose** | **Contents, daily dose** | | | | | |
| **Flavanol** | | | | | | | | | | |
| Calzavara-Pinton 2019 Italy | n=16; 90% F | 26–62 | Healthy，Fitzpatrick skin phototypes I or II | 4–6g （1 g of cocoa powder with 219.6 mg of polyphenols, 55.1 mg of total flavanols, 23.32 mg of epicatechin, 12.15 mg of catechin, 5.1 mg of caffeine, 29.3 mg of theobromine, and 1.36 mg of theophylline）; | 1 g （219.6 mg of polyphenols, 55.1 mg of total flavanols, 23.32 mg of epicatechin, 12.15 mg of catechin, 5.1 mg of caffeine, 29.3 mg of theobromine, and 1.36 mg of theophylline） | tablets | 1w | 22–22.5;43–45 | Forearms | AE = 0 |
| Heinrich 2006 Germany | n=24; 100% F | 18-65 | Healthy，skin phototypes II | 329 mg of cocoa flavanols | 27 mg of cocoa flavanols | beverage | 12w | NR | Back and scapular region | NR |
| Mogollon 2014; Canada | n=59; 100% F | 20–65 | Non smoking healthy women，Fitzpatrick skin phototypes I or II | 30 g chocolate（600 mg of flavanols） | 30 g chocolate （＜90mg of flavanols） | chocolate | 12w | 20±2; 40-60 | Arm | AE = 0 |
| Williams 2009 UK | n=30; 73% F | 42.7 ± 10 | Healthy，Fitzpatrick skin phototypes II and III | 20 g portion of chocolate（＞600mg of flavanols） | 20 g portion of chocolate（＜30mg of flavanols） | droplets | 12w | NR | Forearm | NR |
| Yoon 2016 Korea. | n=62; 100% F | ≥40 （＜ 75） | Healthy female volunteers with visible wrinkles ≥ grade 2 | a cocoa beverage（320 mg flavanols） | a nutrient-matched cocoa-flavored beverage without cocoa flavanols | beverage | 24w | 20–22; 45–55 | Facial and buttock | AE = 0 |
| **carotenoids** | | | | | | | | | | |
| Baswan 2020 USA | n=58; 72% F | 20-40 | generally healthy subjects, Fitzpatrick types II-IV | the multi-carotenoid supplement(taken three times daily and contained β-carotene 4.25 mg, α-carotene 1.10 mg, lutein 1.12 mg, and zeaxanthin 0.053 mg per softgel and other inert ingredients) | Placebo(identical to the Multi Carotene supplement but did not contain any active ingredients). | softgel | 12w | NR | the mid to lower back | AE=0 |

| | | | | | | | | | | |
|---|---|---|---|---|---|---|---|---|---|---|
| Carrascosa 2017<br>German | n=42<br>83%F | 18-60 | The healthy(Fitzpatrick skin phototypes II or III) | The active formulation (Genosun oral®) contained a combination of astaxan thin (4 mg), β -carotene (4.8 mg), vitamin E (6 mg), vitamin C (40 mg), lutein (2.4 mg) and lycopene (2.4 mg). | a pill<br>devoid of the active ingredient but identical in all other respects to the trial product | tablet | 56d | NR | back | NR |
| Cho 2010<br>Korea | n=29<br>100%<br>F | 49-68 | Healthy female | 90 mg of β -carotene | 30 mg of β -carotene | soft capsule | 90d | 20–25 ° C<br>45–55% | facial | AE=0 |
| Bouilly-Gauthier 2010<br>France | n=43<br>100%<br>F | 18- | healthy women（skin type III or IV.） | 5 · 108 colony-forming units<br>of La1 (Skin-ProbioticTM) and 7Æ2 mg carotenoids | maltodextrin | NR | 6W | NR | back | NR |
| Heinrich 2003<br>Germany | n=24<br>67%<br>F | 22-55 | healthy adults(skin type II ) | 24 mg of β-carotene with soybean oil as the vehicle | soybean oil. | soft gel capsule | 12W | NR | back, scapular region) | NR |
| **Lycopene** | | | | | | | | | | |
| Groten 2019<br>Germany，UK | n=149<br>77%<br>F | 20-50 | The healthy(Fitzpatrick skin phototypes I or II) | 15 mg lycopene, 5.8 mg phytoene and phytofluene, 0.8 mg β-carotene,<br>5.6 mg tocopherols from tomato extract, and 4 mg carnosic<br>acid | placebo made from medium-chain triglycerides | soft gel capsule | 12W | NR | buttock | AE=0 |
| Rizwan 2011<br>U.K. | n=17<br>100%<br>F | 21–47 | healthy women(phototypes I or II) | 55 g tomato paste (16 mg lycopene) in olive oil | olive oil alone | paste | 12W | NR | upper<br>buttock | AE=0 |
| Aust 2005<br>Germany | n=24 | NR | healthy adult | 10.2mg lycopene | 8.2mg lycopene | capsules | 12W | NR | back | NR |
| Stahl 2001<br>Germany | n= 22<br>64%<br>F | 26–67 | Healthy adults (skin type II) | 40 g tomato paste with 10 g olive oil, providing ;16 mg lycopene, 0.5 mg b-carotene and 0.1 mg lutein | 10 g olive oil/ | paste | 10W | NR | scapular region | AE=0 |
| **Astaxanthin** | | | | | | | | | | |
| Ito 2018<br>Japan | n=22<br>91%<br>F | 30-60 | The healthy (skin type II or type III) | 4 mg of astaxanthin | a filling<br>agent instead of astaxanthin | capsule | 9W | 21 ± 1◦C<br>50 ± 5% | back | AE=0 |

| | | | | | | | | | | |
|---|---|---|---|---|---|---|---|---|---|---|
| Yoon 2014<br>Korea | n=44<br>100%<br>F | 41-60 | healthy female | 960 mg of medium chain triglycerides ,40 mg of dark red lipid extract and 2 mg of astaxanthin | 960 mg of medium chain triglycerides ,40 mg of dark red lipid extract | capsules | 12W | 20–25C<br>45–55% | cheek | AE=0 |
| Tominaga, 2012<br>Japan | n=36<br>0%<br>F | 20-60 | healthy<br>men | canola oil and 3 mg of astaxanthin. | only canola oil | soft gel capsules | 6W | 20±2°C<br>45±10% | cheek | NR |
| Collagen | | | | | | | | | | |
| Lee 2023<br>Korea | n=100<br>100%<br>F | 30-60 | mentally and physically healthy, had dry facial skin | CPNS 1650mg, Cellulose 1485mg, Excipients 132mg | CPNS 0mg, Cellulose 3135mg, Excipients 132mg | tablet | 12W | 22 ± 2 °C<br>50 ± 5% | Cheek | AE=0 |
| Sangsuwan 2020<br>Thailand | n=36<br>100%<br>F | 50-60 | healthy Thai women | 5g Collagen supplement | Maltodextrin | packet | 4W | NR | Cheek | AE=0 |
| Miyanaga 2021<br>Japan | n=66<br>100%<br>F | 35–50 | healthy women | The test beverage 50 mL (containing 1 or 5 g of CP) | placebo beverage 50 mL | beverages | 12W | 21 ± 2°C<br>45% ± 5% | cheek | AE=0 |
| Lin 2021<br>Taiwan | n=50<br>100%<br>F | 35-50 | Healthy women | collagen drink 50 g; main ingredient: 81% water, 11% fish collagen (extracted from Pangasius hypophthalmus, Oreochromis niloticus, Clarias gariepinus, Gadus morhua, Ictalurus furcatus, and Melanogrammus aeglefinus), 3% apple juice, 2% Djulis extract | placebo drink 50 mL; main ingredient: 95% water, 3%apple juice | drink | 8W | NR | upper cheek | NR |
| Evans 2021<br>Canada | n=36<br>100%<br>F | 45-60 | Healthy women | 10 g of hydrolyzed collagen | 10g placebo powder | powder | 12W | NR | cheek | AE=1 |
| Žmitek 2020<br>Slovenia | n=34<br>100%<br>F | 40-65 | healthy Caucasian female | 10 mL of a syrup: hydrolysed fish collagen: 4000 mg, water-soluble CoQ10, 50 mg, itamin C:80 mg, vitamin A: 920 µg, biotin: 150 µg | 10 mL of flavoured and coloured placebo syrup without any active ingredients | syrup | 12W | 20–25 ◦C<br>40–60%, | cheek | AE=0 |
| Bolke 2019<br>Germany | n=72<br>100%<br>F | 35-73 | healthy female | 2.5 g collagen peptides, 666 mg acerola fruit extract, 80 mg vitamin C, 3 mg zinc, 2.3 mg vitamin E, and 50 µg biotin | potassium sorbate, sodium benzoate,arboxymethylcellu lose,citric acid, natural aroma, and water,and did notcontain any nutrients | NR | 12W | 20 ◦C<br>40%–60% | forearm | AE=0 |

| | | | | | | | | | | |
|---|---|---|---|---|---|---|---|---|---|---|
| Kim 2018<br>Korea | n=53<br>100%<br>F | 40–60 | healthy female | Low-molecular-weight Collagen peptide 1000mg, Vitamin C 100mg, Fruit concentrate mix 3000mg, | Low-molecular-weight Collagen peptide 0mg, Vitamin C 100mg, Fruit concentrate mix 3000mg, | Bottle (liquid supplement ) | 12W | 40–60%<br>22–24 ◦C | crow’s-feet area | AE=0 |
| Czajka 2018<br>Italy | n=120<br>76%<br>F | 21-70 | healthy female or male | hydrolyzed fish collagen type I (4,000 mg), molecular weight of 0.3–8 kDa, hyaluronic acid, glucosamine hydrochloride, L-arnitine, black pepper and maca extracts | The placebo contains water (which replaces the active ingredients present in the test product), natural preservatives (malic acid and citric acid), flavoring (mango and apple) and a natural sweetener (stevia). | Bottle (liquid supplement ) | 90D | NR | forearm, | AE=0 |
| Inoue 2016<br>China | n=58<br>100%<br>F | 35-55 | healthy Chinese female s | collagen hydrolysate (H-CP) had dipeptide-to-product content, with more than 2 g kg−1 of product | maltodextrin TK-16, | aluminium sachet | 8W | 20±2◦C<br>50±5% | Cheek | AE=0 |
| Asserin 2015<br>Japan | n=22<br>100%<br>F | 40-59 | healthy Japanese women | specific collagen peptides of fish origin and porcine origin with an average molecular weight of 2000–5000 Da | dextrin | drink | 8W | 22±1 °C<br>50 ±10% | Cheek | NR |
| Proksch 2014<br>Germany; | n=110<br>100%<br>F | 45-65 | healthy female subjects | 2.5 g BCP composed of different specific collagen peptides,an average molecular weight of 2.0 kD. | maltodextrin | powder | 8W | 21.5°C<br>50% | lateral canthus | AE=0 |
| Choi 2014<br>Korea | n=16<br>75%<br>F | 30-48 | healthy female | 3 g 15% tripeptide form, highly advanced-collagen tripeptide, Average molecular weight was 1500Da. | no supplement | NR | 12W | NR | NR | AE=0 |
| Genovese 2017<br>Italy | n=120<br>93%<br>F | 40-60 | healthy female and male | hydrolyzed collagen type I (5,000 mg), with a molecular weight of 0.3–8 kDa, hyaluronic acid, borage oil and N-acetylglucosamine | Water, stevia, peach and lychee flavoring , citric acid, malic acid, soybean polysaccharide | beverages | 90D | NR | forearm | AE=0 |
| hyaluronan | | | | | | | | | | |
| Michelotti 2021<br>Italy | n=60<br>100%<br>F | 35-70 | healthy female | 200-mg of FSHA | maltodextrin | drink | 28D | 22 ± 2 ◦C<br>40-60% | cheek | AE=0 |

| | | | | | | | | | | |
|---|---|---|---|---|---|---|---|---|---|---|
| Hsu 2021 Taiwan. | n=41 63% F | 35-64 | healthy Asian men and women | 120 mg HA | dextrin; | capsule | 12W | 20 ± 2◦C 50 ± 5% | Arm | AE=0 |
| Oe 2017 Japan | n=35 57% F | 22-59 | healthy Japanese male and female | 60 mg HA with microcrystalline cellulose, total 210 mg per capsule | 210 mg microcrystalline | capsule | 12W | 22 ± 2°C 50% ± 15% | corner of eye | AE=0 |
| Kawada 2015 | n=42 100% F | 35-60 | healthy Japanese female | HA (120 mg) was mixed with 420 mg of hydrogenated maltose | 270 mg of cellulose | capsules | 6W | 21 ± 2°C 50 ± 10% | cheek | NR |
| polyphenol | | | | | | | | | | |
| A Nobile 2021 Italy | n=50 100% F | 35–65 | Asian female | 250 mg maltodextrin(four polyphenolic Extracts) and 120 mg microcrystalline | 250 mg maltodextrin and 120 mg microcrystalline cellulose | capsules | 12W | NR | cheek | AE=0 |
| C Nobile 2021 Italy | n=50 100% F | 35–65 | Caucasian female | 250 mg maltodextrin(four polyphenolic Extracts) and 120 mg microcrystalline | 250 mg maltodextrin and 120 mg microcrystalline cellulose | capsules | 12W | NR | cheek | AE=0 |
| Shoji 2020 Japan | n=38 100% F | 20-39 | Healthy women with skin photo-type-II and -III | 600 mg of Apple Polypheno | 600 mg starch decomposition product | tablets | 12W | 20 ± 1◦C 45 ± 5% | upper arm | AE=0 |
| Heinrich 2011 Germany | n=60 100% F | 40-65 | Healthy female | 1 L of the green tea beverage(1402 mg total tea catechins) | 1 L/d of a constituent matched beverage. | beverage | 12W | 21 ± 1◦C 40% | forearm | NR |
| Janjua 2009 USA | n=56 100% F | 25-75 | healthy female v | 250 mg polyphenols | The placebo capsules were identical in appearance to the green tea capsules. | capsules | 2Y | NR | Arm | AE=0 |

F = female; NR = not reported; AE = adverse events; it = intervention; pl = placebo; TAE = treatment-related adverse effect; TWD = treatment-related withdrawals;

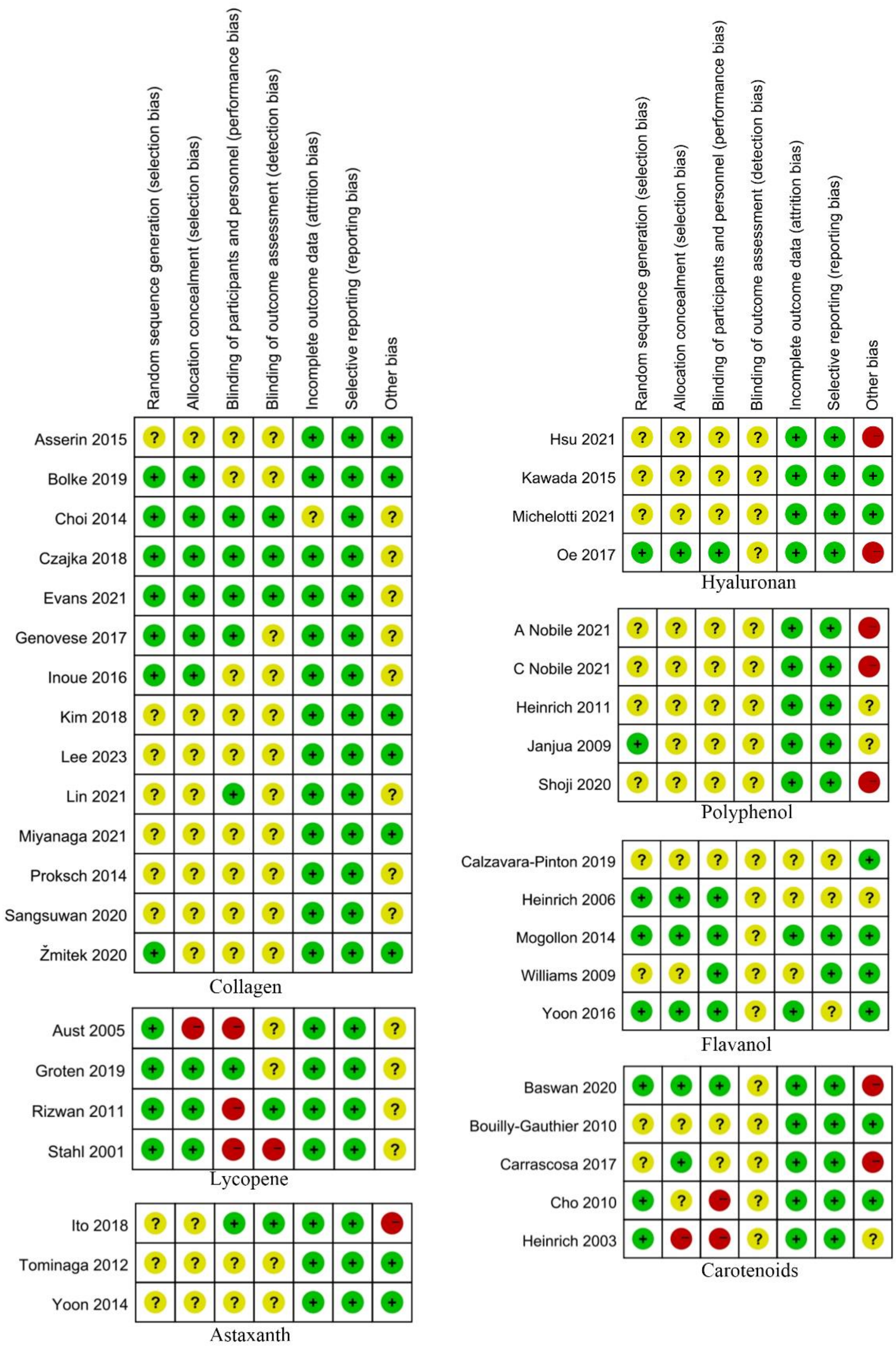


**Supplemental Fig.S1** Risk of bias summary: review authors' judgements about each risk of bias domain for each included study. + = low risk of bias; ? = unclear risk of bias; - = high risk of bias
